# Drying kinetics, power consumption, economic and environmental analysis of pomegranate peels drying using a hybrid solar dryer compared with oven dryer

**DOI:** 10.1038/s41598-025-22464-7

**Published:** 2026-02-13

**Authors:** Khaled A. Metwally, El-Sayed G. Khater, Adel H. Bahnasawy, Aml Abubakr Tantawy, Ahmed Elbeltagi, Ali Salem, Samy A. Marey, Abdelaziz M. Okasha, Khaled Abdeen Mousa Ali, Abdallah Elshawadfy Elwakeel

**Affiliations:** 1 Soil and Water Sciences Department, Faculty of Technology and Development, Zagazig University, Zagazig, 44519 Egypt; 2 Agricultural and Biosystems Engineering Department, Faculty of Agriculture, Benha University, P.O. Box 13736, Moshtohor, Toukh, Kalubia Egypt; 3 Food Science Department, Faculty of Agriculture, Beni-Suef University, Beni-Suef, 65211 Egypt; 4 Agricultural Engineering Department, Faculty of Agriculture, Mansoura University, Mansoura, 35516 Egypt; 5 Civil Engineering Department, Minia University, Minia, Egypt; 6 Structural Diagnostics and Analysis Research Group, Faculty of Engineering and Information Technology, University of Pécs, Pecs, Hungary; 7 Department of Agricultural Engineering, College of Food and Agriculture Sciences, King Saud University, 11451 Riyadh, Saudi Arabia; 8 Department of Agricultural Engineering, Faculty of Agriculture, Kafrelsheikh University, Kafr El-Sheikh, 33516 Egypt; 9 College of Engineering, South China Agricultural University, Guangzhou, 510642 China; 10 Agricultural Engineering Department, Faculty of Agriculture and Natural Resources, Aswan University, Aswan, Egypt

**Keywords:** Carbon footprint, Carbon mitigation, Renewable energy, Suitability, Healthy foods, Postharvest technology, Plant sciences, Environmental sciences, Energy science and technology

## Abstract

Drying pomegranate peels, a by-product of juice production, preserves their beneficial properties and minimizes waste. Using optimal drying conditions, such as controlled temperatures and thin layers, improves efficiency and ensures high quality. These dried peels can then be utilized in various industries, including food, pharmaceuticals, and cosmetics. To our knowledge, there are no existing studies that detail the effects of hybrid solar drying, drying temperatures, and layer thickness on the drying kinetics, power consumption, and economic and environmental aspects. In this study, a hybrid indirect SD (HISD) with a temperature and humidity control unit was used to dry pomegranate peels at three different temperatures—50 °C, 60 °C, and 70 °C—and three different thicknesses—1, 2, and 3 cm. The HISD was then compared to a conventional oven drying system (CODS). The obtained results indicated that increasing the drying temperature increased the weight loss of pomegranate peels. Also, the average initial moisture content of pomegranate peels was 76.5% (w.b.). The final MC ranged from 2.67 to 2.10% and from 2.97 to 2.84% for the CODS and HISD, respectively. The higher drying rates of the pomegranate peels dried using CODS and HISD were 169.79 and 196 kg_water/kgdrymatter_/h, respectively, at a layer thickness of 3 cm and a drying temperature of 70 °C. Additionally, using HISD led to a reduction in power consumption by about 64.28% to 75.75% compared to the CODS. Furthermore, the environmental analysis results showed that the embodied energy is about 1270.463 kW.h. The energy payback period for HISD ranges between 2.38 and 6.34 years. The earned carbon credit for drying pomegranate peels using the HISD ranged between 770.1 and 2207.2 USD. Based on economic analysis, the lowest drying cost using the HISD was 144.5 USD per ton of pomegranate peels, achieved at layer thicknesses of 1 cm and a drying temperature of 70 °C.

## Introduction

Solar dryers (SDs) represent an advanced and environmentally sustainable technology for drying agricultural products and food byproducts, providing a greener alternative to conventional drying techniques^1–5^. By utilizing solar energy as their primary heat source, these systems significantly reduce dependence on fossil fuels, cut energy expenses, and lessen the negative environmental impact associated with traditional drying methods^6,7^. Solar dryers are especially advantageous in areas that receive plentiful sunlight, where they can effectively dry a wide range of materials—including crops, fruits, vegetables, herbs, and various agricultural residues—while maintaining the nutritional and sensory qualities of the dried products and prolonging their storage life^7–9^. Furthermore, the adoption of solar drying technologies supports sustainable agricultural practices, promotes rural development by offering cost-effective and accessible drying solutions, and contributes to reducing post-harvest losses, which are a major challenge in many developing regions^3,4,10,11^.

SDs operate by converting sunlight into thermal energy, which is used to remove moisture from the products^12–17^. They come in various designs, including direct, indirect, and hybrid dryers^12,18,19^. Direct SDs expose the product to sunlight, while indirect dryers use solar-heated air to dry the product in a separate chamber^20,21^. Hybrid dryers combine solar energy with auxiliary heating sources for consistent performance in varying weather conditions^22–26^. These systems are cost-effective, easy to maintain, and adaptable to small-scale and large-scale operations^21,23^.

The use of SDs addresses key challenges in post-harvest management, such as spoilage, mold growth, and nutrient degradation caused by improper drying^22,27,28^. By maintaining optimal drying temperatures, SDs ensure better product quality and reduce post-harvest losses^27,29^. Additionally, they contribute to food security and economic sustainability by enabling farmers to process and store surplus produce for off-season use or market sale^30,31^.

The pomegranate fruit (*Punica granatum L*.) market size was valued at USD 248.4 Million in 2023 and is poised to grow from USD 261.57 Million in 2024 to USD 356.55 Million by 2032, growing at a CAGR of 5.3% during the forecast period (2025–2032)^32^. Egypt, Spain and Israel are the most prominent exporting countries, but the Egyptian crop is of great importance in international contracts due to its high quality and the organization of the export process. The high demand for Egyptian pomegranates has contributed to raising its prices to record levels, especially in the markets of Europe, Russia and Arab countries, most notably Iraq^33^. Pomegranate peels, typically considered agricultural byproducts, are a rich source of bioactive compounds such as polyphenols, flavonoids, and tannins, which exhibit potent antioxidant, anti-inflammatory, and antimicrobial properties^34–36^. These peels, often discarded during processing, hold significant potential for valorization in various industries, including food, pharmaceuticals, and cosmetics^37,38^. Processing pomegranates results in considerable amounts of byproducts, mainly peels and seeds, with peels making up around 50% of the fruit’s fresh weight^36^. By drying and processing pomegranate peels, they can be transformed into value-added products like natural preservatives, dietary supplements, or skincare ingredients, contributing to waste reduction and sustainable resource utilization^39,40^.

Drying pomegranate peels using a hybrid SD is an energy-efficient and sustainable approach that addresses power consumption, economic viability, and environmental impact^6,41^. Traditional drying methods, such as open sun drying or conventional electric dryers, are either energy-intensive or inconsistent, leading to high operational costs and environmental concerns^13,42,43^. Hybrid SDs, which combine solar energy with auxiliary heating systems, offer a balanced solution by reducing reliance on non-renewable energy sources while ensuring consistent drying performance^22,44^.

The analysis of energy, economic, and environmental aspects of a hybrid SD is essential for advancing sustainable technologies in food preservation and agriculture^45–48^. Energy analysis focuses on evaluating the system’s efficiency, optimizing the use of solar energy, and integrating auxiliary energy sources to ensure consistent performance. This helps reduce reliance on non-renewable energy and enhances operational reliability^41,49–52^. Economic assessment examines the initial investment, operational costs, payback period, and overall cost-effectiveness, making the technology accessible for both small-scale farmers and industrial users^53–55^. Environmental analysis highlights the system’s role in reducing greenhouse gas emissions, minimizing fossil fuel dependency, and promoting eco-friendly practices. Together, these analyses provide a holistic understanding of the hybrid SD’s viability, ensuring it meets energy-saving goals, economic feasibility, and environmental sustainability. By balancing these factors, hybrid SDs contribute to global efforts in clean energy adoption, food security, and climate change mitigation^56–58^. A hybrid SD combines solar energy with auxiliary heating, offering an eco-friendly alternative to industrial dryers^59,60^. By utilizing renewable solar power, it significantly reduces reliance on fossil fuels, lowering carbon emissions^61,62^. Unlike energy-intensive industrial dryers, hybrid SDs are cost-effective, sustainable, and ideal for small-scale operations^18,63–66^. They preserve product quality while minimizing environmental impact, making them a crucial tool for achieving low-carbon footprints in agriculture and food processing industries. This innovation aligns with global sustainability goals and promotes energy efficiency^44^.

Several researchers and academics have previously investigated the drying of pomegranate peels. For example, Wanderley et al.^67^ examined how drying temperature influences the drying kinetics and physicochemical properties of pomegranate peels, using a hot air circulation oven at 50, 60, and 70 °C. Cecchi et al.^68^ explored the impact of industrial drying processes on the phenolic and polysaccharide content of pomegranate peels to facilitate reuse of by-products, drying peels in an oven at various temperatures while considering peel thickness and size; peel pâté was freeze-dried and oven-dried at 50–110 °C at laboratory scale, then compared with industrial drying at 150 °C. Mphahlele et al.^69^ assessed how different drying methods affect the bioactive compounds and antioxidant, antibacterial, and antityrosinase activities of pomegranate peels, using freeze-drying and oven drying at 40, 50, and 60 °C. Additionally, Mphahlele et al.^70^ investigated the drying kinetics of pomegranate peel dried at 40, 50, and 60 °C with a constant air velocity of 1.0 m/s. Galaz et al.^71^ studied the drying kinetics of pomegranate peels using double drum drying at 100, 110, and 120 °C with a fixed 2 mm clearance, also evaluating the impact on polyphenolic compounds. Finally, Marchi et al.^72^ examined the antioxidant and antimicrobial properties of pomegranate peels subjected to different drying methods, including freeze-drying and oven drying to 60 °C.

SDTo the best of our knowledge, there are no existing studies that comprehensively investigate the combined effects of drying methods, drying temperatures, and thin-layer thickness on the drying kinetics, energy consumption, economic feasibility, and environmental impact of pomegranate peel drying using a Hybrid Indirect SD (HISD). This research introduces a novel integration of an HISD system equipped with a control unit for temperature, relative humidity, and an auxiliary electrical heater to ensure optimal drying conditions. By comparing the performance of this advanced solar-assisted system with a Conventional Oven Drying System (CODS) across varying temperatures and layer thicknesses, the study offers a multifaceted assessment of both systems. The key contributions of this work lie in its holistic approach to evaluating drying performance, its detailed techno-economic and environmental impact assessment, and its practical implications for sustainable agro-waste valorization. This makes the study a valuable addition to the body of knowledge on efficient and sustainable drying technologies for agricultural by-products.

## Materials and methods

### 2.1. Experimental procedures

The research focused on drying fresh pomegranate peels using two distinct drying methods: a SDHISD equipped with an electric heater and a CODS. The pomegranate peels, sourced from local markets in Benha, Egypt, were cleaned, cut, and prepared for drying, with an initial MC of 76.5% (w.b.). The experiments were carried out at the Faculty of Agriculture, Moshtohor, Benha University, Egypt, between May and August 2024. During this period, the ambient conditions varied, with temperatures ranging from 21.6 to 34.1 °C, relative humidity between 42.6 and 77.3%, and solar radiation levels from 321.9 to 954.1 kJ m⁻^2^day⁻^1^. The study examined the effects of three drying temperatures (50, 60, and 70 °C) and three peel layer thicknesses (1, 2, and 3 cm) on the drying process, using a split-plot experimental design with three replicates.

### 2.2. Description of the HISD

**Fig. 1:**
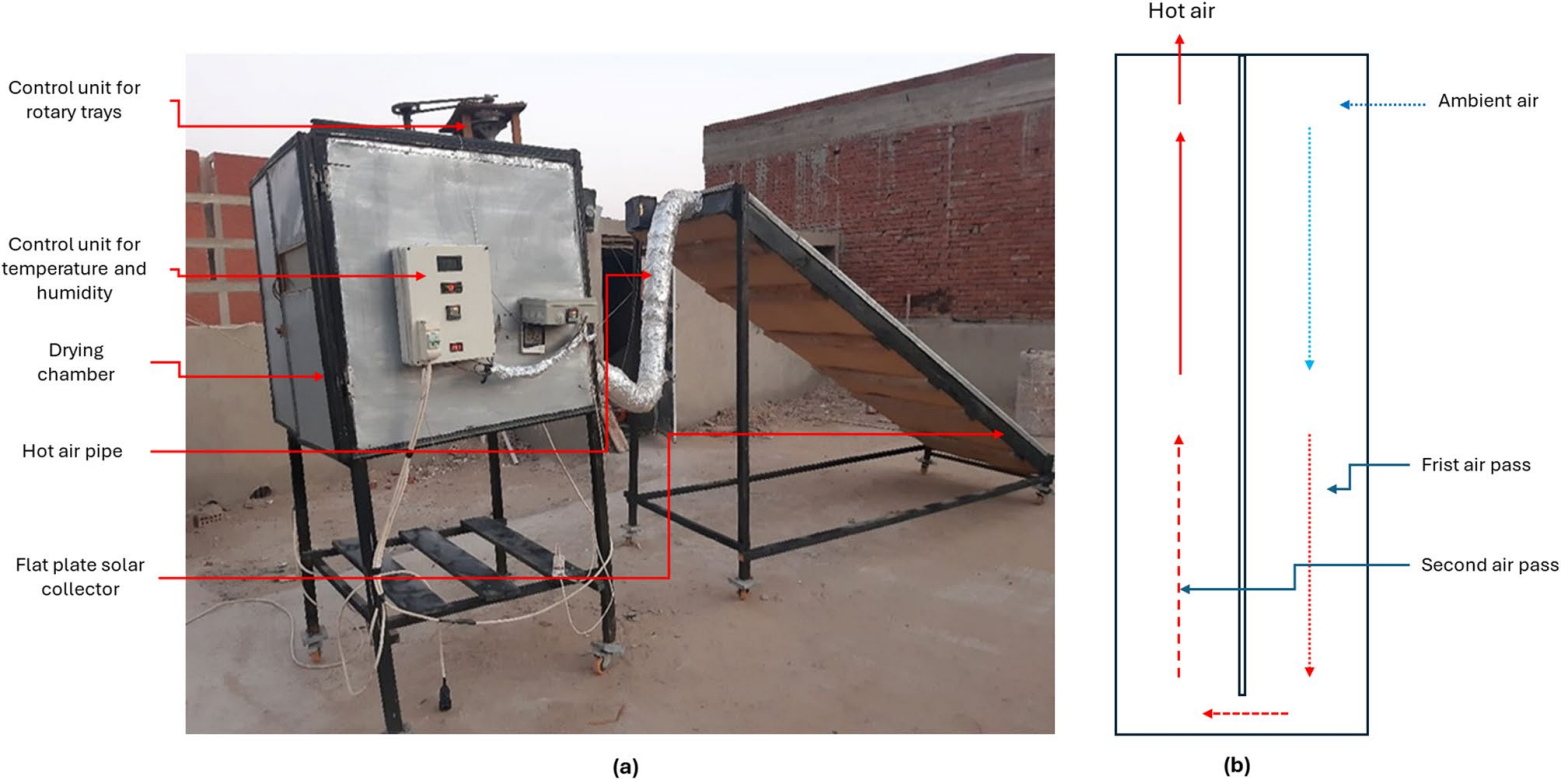
Main components of the HISD. Whereas (**a**). real photo of the HISD, (**b**). double air circulation passes inside the flat plate solar collector.

**Fig. 2:**
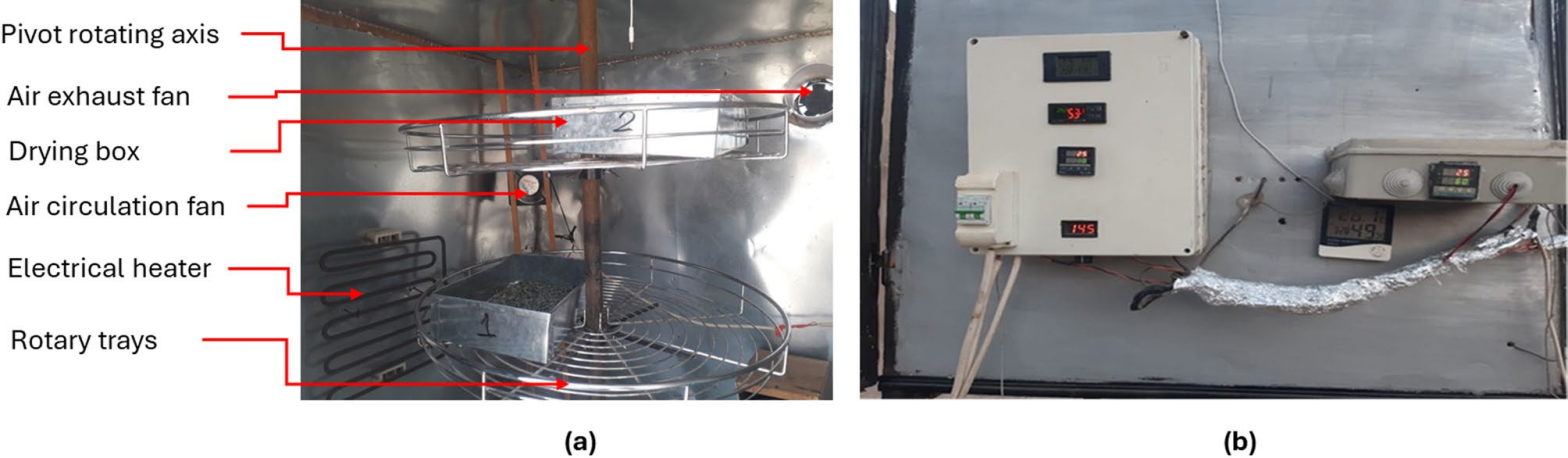
Internal components and control units of the HISD. Whereas, (**a**). internal components of the drying room, and (**b**). control units for temperature, humidity and electrical heater.

The SDHISD was developed and utilized to meet the objectives of the current study. It incorporates a double air pass flat plate solar collector and is supplemented by an electric heater to regulate and increase the air temperature inside the drying chamber. The HISD (Fig. 1) consists of several key components: 1. Solar collector: Measuring 4 m in length, 1 m in width, and 20 cm in depth, it is covered with a 3 mm thick glass layer; 2. Absorber Plate: A corrugated black aluminum plate serves as the absorber, insulated with 5.0 cm thick thermal wool; 3. Drying Chamber: Constructed from 5 mm thick galvanized steel, it has dimensions of 1.0 m (length), 1.0 m (width), and 1.0 m (height). The walls are insulated with 2 cm of thermal wool and 3 cm of foam; 4. Drying Boxes: Made of stainless steel, these boxes measure 30 cm in length, 20 cm in width, and 7 cm in height. They feature perforated bottoms to allow heated air to flow through the drying products; 5. Rotary Trays: The drying chamber includes two rotary trays (Fig. 2a), each 60 cm in diameter and 3 cm in height, mounted on a pivoted rotary axis.; 6. Electric Heater: A 2000 W electric heater serves as an auxiliary heating system, enhancing the air temperature and reducing relative humidity inside the chamber to speed up the drying process; 7. Air Fans: Two fans (Model C.C.P. Parm, Italy) with a flow rate of 6.6 m^3^/h, 2800 rpm, and 150 W power were used to force hot air from the solar collector into the drying chamber and circulate it internally; 8. Control Systems: Two control systems were employed. The first is a digital controller (model: REX_C100 PID), which adjusts the temperature and humidity by regulating the electric heater and fan speed. The second system manages the air fans to maintain optimal drying conditions within the chamber (Fig. 2b); 9. Main frame: it was manufactured from wood only to reduce the total manufacturing costs, reducing the total weight of the SD and reducing the EE and carbon footprint of the SD. This setup ensures efficient and controlled drying, combining solar energy with auxiliary heating for consistent performance. Table 1 shows the specifications of instruments used.

**Table 1:** Specifications of instruments used.

Instrument/component	Model/Part No	Manufacturer	Measurement range/function	Accuracy/resolution	Country of origin
PID temperature controller	REX-C100FK02-M*DA	RKC instrument Inc	0–400 °C, controls heater and fan via relay or SSR	± 0.5% o	Japan
Thermocouple Probe (K-type)	Standard K-type	Omega engineering	–50 to 400 °C	± 1.5 °C	USA
Humidity & temperature sensor	SHT31	Sensirion AG	–40 to + 125 °C; 0–100% RH	± 2% RH; ± 0.3 °C	Switzerland
Data logger	ValProbe RT	Kaye instruments	0–70 °C; 15–95% RH	0–70 °C; 15–95% RH	USA

### 2.3. Drying kinetics

#### 2.3.1. Moisture content (MC)

To estimate the initial MC in a laboratory, the procedure outlined by AOAC^73^, is followed, and the oven-drying method is commonly employed. First, an empty, dry container is weighed (W₁). The sample (pomegranate peels) is added, and the total weight is recorded (W₂). The sample is then dried in an oven at 105 °C until a constant weight is achieved. After drying, the sample is cooled in a desiccator to prevent moisture absorption. Finally, the container with the dried sample is weighed (W₃), and the MC is calculated using Eq. 1.

1 $$M_{C} \left( \% \right) = \left[ {\frac{{W_{2} - W_{3} }}{{W_{2} - W_{1} }}} \right] \times 100$$

#### 2.3.2. Accumulated weight loss

Weight loss is calculated using Eq. 2^74^^.^

2 $$Weight loss \left( g \right) = W_{t} - W_{t + 1}$$

#### 2.3.3. Drying rate

The drying rate is a critical parameter in drying processes as it directly influences the efficiency, quality, and energy consumption of the operation. It measures how quickly moisture is removed from a material, such as pomegranate peels, and is essential for optimizing drying conditions like temperature, airflow, and layer thickness. A faster drying rate reduces processing time, enhancing productivity and minimizing energy costs. However, excessively high drying rates can lead to quality degradation, such as cracking, nutrient loss, or uneven drying. Conversely, a slow drying rate may preserve quality but increase energy use and risk microbial growth. Understanding the drying rate helps in designing efficient drying systems, selecting appropriate drying methods, and ensuring the preservation of the material’s nutritional, structural, and sensory properties. It also aids in predicting drying time, improving process control, and reducing operational costs, making it vital for industries like agriculture, food processing, and pharmaceuticals. The drying rate was calculated using Eq. 3, as described by Etim et al.^75^.

3 $$Drying rate \left( {g_{water} .g_{dry matter}^{ - 1} h^{ - 1} } \right) = \frac{Weight loss \left( g \right)}{{\Delta t \left( h \right)}}$$

### 2.4. Environmental analysis

In recent years, researchers have intensified efforts to reduce total energy consumption—including both electrical and fossil fuel energy—in constructed buildings. The goal is to minimize reliance on non-renewable energy sources and promote sustainability. SDs, for instance, are an excellent example of energy-efficient systems that harness freely available solar energy, a clean and renewable resource^57,76^. The energy efficiency of such systems can be assessed by analyzing their total energy consumption. Furthermore, their environmental impact can be evaluated using key indicators such as EE, energy payback period, and greenhouse gas emissions throughout their life cycle. These analyses are essential for developing sustainable technologies that align with global energy conservation and environmental protection goals.

The specific energy consumed (SEC) for drying refers to the amount of energy required to remove a unit mass of moisture, typically water, from a material during the drying process. And the Specific energy consumed for drying pomegranate peels is obtained by the following Eq. 4 according to Fudholi et al.^77^.

4 $$E_{specific} = \frac{{E_{total} }}{{m_{water} }}$$

where ${E}_{total}$ is Total energy consumed during the drying process in kW.h), and ${\mathrm{m}}_{\mathrm{water}}$ is Mass of water removed (in kg).

In the case of the use of HISD for drying the amount of energy required to remove a unit mass of moisture is sum of solar energy and electrical energy.

5 $$E_{total} = E_{solar} + E_{elctrical}$$

where, ${E}_{solar}$ is the solar energy collected by the solar collector in kW.h, and ${E}_{elctrical}$ is the electrical power consumed by the electrical heater in kW.h.

The solar energy collected by the solar collector (${E}_{solar} in J$) is estimated as mentioned by Usub et al^78^,

6 $$E_{solar} = A_{c} \mathop \smallint \limits_{0}^{t} Ins_{c} \left( t \right)dt$$

where, ${A}_{c}$ is the surface area of the solar collected in m, and ${Ins}_{c}$ is the solar intensity in W m^-2^.

The energy output from water evaporation in a SD is derived from the latent heat of vaporization required to convert liquid water into vapor. As the dried product loses moisture, energy is absorbed from the surrounding air within the dryer. This process is influenced by factors like solar radiation, air temperature, and humidity. Efficient evaporation ensures effective drying, reducing MC while utilizing solar energy, making it a sustainable method for preserving agricultural products. The following is the energy output (${E}_{output} in kJ$) from the solar air collector according to Balal et al.^79^,

7 $$E_{output} = \mathop \smallint \limits_{0}^{t} \dot{m}\left( t \right) \times C_{pa} \left( {T_{c} - T_{in} } \right) dt$$

where, m is the mass air flow rate in kg/s, ${C}_{pa}$ is the air’s specific energy, expressed in kJ kg^−1^ k^−1^, and ${T}_{c}-{T}_{in}$ is the temperature differential in k.

The EE of a HISD (Table 2) refers to the total energy consumed during its lifecycle, including raw material extraction, manufacturing, transportation, assembly, and maintenance. SDs typically have lower EE compared to conventional dryers, as they rely on renewable solar energy for operation rather than fossil fuels. Materials like metals, glass, and insulation contribute to the EE, but their impact is offset by the dryer’s energy-efficient and sustainable performance over its lifespan.

**Table 2:** EE calculation data for construction the HISD^60,80,81^.

No	Materials	EE (kW.h/kg)	Weight (kg)	EE (kW.h)
Solar collector				
1	Wooden frame	8.5	35	297.5
2	Glass cover	7.28	10	72.8
3	Thermal wool	16	2.0	32
4	Paint	25.11	1.0	25.11
5	Absorber plate	38.5	10	385
Drying room				
1	Wooden frame	8.5	25	212.5
2	Thermal wool + foam	2.0	2.0	4
3	Paint	25.11	0.5	12.56
4	Hinges	55.28	0.05	2.764
	Handel	55.28	0.05	2.764
5	*Suction fan*			
	1. Plastic parts	19.44	0.30	5.832
	2. Motor and cooper wires	19.61	0.30	5.883
6	Drying trays	38.5	5.0	192.5
7	Electrical heater	38.5	0.5	19.25
Total EE (EE) for the HISD = 1270.463 kW.h				

Energy payback time is defined as the time needed to repay the EE of the HISD and estimated using Eq. 8, as mentioned by Prakash et al.^82^,

8 $${\text{Time of energy payback}},{\text{ year}} = \frac{{{\text{EE }}\left( {{\mathrm{kW}} \cdot {\mathrm{h}}} \right)}}{{{\text{Yearly energy output }}\left( {{\mathrm{kW}} \cdot {\mathrm{h}}} \right)}}$$

The yearly emission of carbon dioxide (${E}_{CO2}$) can be determined as described by Prakash et al.^82^,

9 $$E_{{CO2}} = \frac{{{\text{EE }}\left( {{\mathrm{kW}} \cdot {\mathrm{h}}} \right) \times 0.98}}{{{\mathrm{lifetime}},{\text{ year}}}}$$

In this study, to lessen emissions of carbon dioxide and reduce carbon footprint, the HISD was designed using a construction material with low EE materials such as wood, where it was used for manufacturing the main frame of the SD. Additionally, the HISD is an innovative and sustainable solution that combines solar energy with auxiliary heating systems, offering a greener alternative to traditional industrial dryers. Unlike industrial dryers, which rely heavily on fossil fuels and consume significant amounts of energy, hybrid SDs harness renewable solar power, drastically reducing carbon emissions. They are particularly beneficial for small- to medium-scale operations, providing cost-effective and energy-efficient drying while maintaining product quality. By minimizing reliance on non-renewable energy sources, HISDs contribute to a lower carbon footprint, supporting global efforts to combat climate change and promoting sustainable practices in agriculture and food processing industries. To assess the effectiveness of the developed drying system, it is necessary to compare it with the traditional system used in an experimental setting at a coal-fired power generation station. These stations generate electricity, which then transmits and distributes to the electric-powered dryers for consumption. Where the average carbon dioxide emission from coal-generated energy is approximately 0.98 kg per kilowatt-hour (kWh); additionally, assuming transmission losses of 40% and 20% due to old appliances.

10 $$The CO_{2} mitigation per kWh of the dryer = \frac{1}{{1 - L_{a} }} \times \frac{1}{{1 - L_{td} }} \times 0.98$$

According to Nayak et al.^54^, the carbon dioxide mitigation throughout lifespan of the HISD is,

11 $$The CO_{2} mitigation over the system lifetime \left( {kg} \right) = E_{EE} \times \frac{1}{{1 - L_{a} }} \times \frac{1}{{1 - L_{td} }} \times 0.98$$

The net mitigation of carbon dioxide over the lifetime of the HISD refers to the total reduction in carbon emissions achieved by using solar energy instead of fossil fuels. By harnessing renewable energy, SDs significantly lower carbon dioxide emissions, contributing to climate change mitigation and promoting sustainable, low-carbon drying practices. Where the net mitigation of carbon dioxide over the lifetime of the HISD was estimated according to Eq. 12,

12 $$\begin{aligned} {\text{Net mitigation over the lifetime }}\left( {{\mathrm{kg}}} \right){\mkern 1mu} = & {\mathrm{TotalCO}}_{2} {\text{mitigation }} - {\text{ Total CO}}_{2} {\mathrm{emission}} \\ = & \left( {E_{{output}} \times n_{{HISD.}} } \right) \times \frac{1}{{1 - L_{a} }} \times \frac{1}{{1 - L_{{td}} }} \times 0.98 - E_{e} \\ \end{aligned}$$

where ${E}_{out}$ is the dryer’s annual thermal output (kWh), ${n}_{HISD.}$ is the lifespan of the HISD (considered as 30 years), and ${E}_{e}$ is an input of the EE (kWh) (Table 2).

The earned carbon credit ($ECC$) corresponds to the reduction of one metric ton (1000 kg) of carbon dioxide emissions, and the credit obtained from the HISD was determined using Eq. 13 as reported by Vijayan et al.^83^.

13 $$ECC = Net mitigation of CO_{2} in life time \times Price per ton of CO_{2} mitigation$$

### 2.5. Economic analysis.

An economic analysis of hybrid SDs is crucial for understanding their financial viability and long-term benefits. By combining solar energy with auxiliary heating, these dryers offer a sustainable and cost-effective alternative to conventional drying methods. The analysis evaluates initial investment, operational savings, and maintenance costs, highlighting the reduction in energy expenses due to the use of renewable solar power. It also considers payback periods and return on investment, demonstrating the economic advantages of hybrid systems. This assessment helps stakeholders make informed decisions, encouraging the adoption of energy-efficient and environmentally friendly drying technologies that align with both economic and sustainability goals^84–86^.

In the current study, the economic analysis of the HISD was performance based on the Egyptian financial conditions. Where the annualized investment cost of hybrid SDs is crucial for assessing affordability and long-term financial benefits, helping stakeholders evaluate cost-effectiveness and make informed decisions about sustainable, energy-efficient drying solutions. The annualized investment cost (${C}_{AN}$) and yearly capital cost (${C}_{CC}$) of the HISD were calculated using Eq. 14 as stated by Yang et al.^87^.

14 $$C_{AN} = (C_{CC} \times F_{CR} ) + C_{M} + C_{EC} - \left( {S \times F_{SF} } \right)$$

where ${F}_{CR}$ is the capital recovery factor, ${C}_{M}$ is the maintenance cost, ${C}_{EC}$ is the electricity cost, $S$ is the salvage value of the HISD, and ${F}_{SF}$ is the salvage fund factor.

The maintenance cost of the HISD was assumed to be 3% of the annualized investment cost. Additionally, the salvage value of the HISD was assumed as 8% of the annualized investment cost. The yearly capital cost (${C}_{ac}$) was estimated according to Eqs. 15 and 16,

15 $$F_{{CR}} = \frac{{d\left( {1 + d} \right)^{{n_{{HISD}} }} }}{{\left( {1 + d} \right)^{{n_{{HISD}} }} - 1}}$$

16 $$F_{{SF}} = \frac{d}{{\left( {1 + d} \right)^{{n_{{HISD}} }} - 1}}$$

where ${C}_{cc}$ is total capital cost, *d* is the interest rate (equal to 15%), and ${n}_{HISD.}$ is the lifetime of the HISD (equal to 30 years).

The drying cost per kilogram of pomegranate peels (${C}_{s}$) and the dried quantity per year (${M}_{y}$) of pomegranate peels using HISD were estimated using Eqs. 17 and 18 as reported by Singh et al.^88^and Yang et al.^89^,

17 $$C_{s} = \frac{{C_{a} }}{{M_{y} }}$$

18 $$M_{y} = \frac{{M_{d} \times D}}{{D_{d} }}$$

where, *D* is the drying days per year, ${M}_{d}$ is the amount of pomegranate peels every batch, and ${D}_{d}$ is the drying time per batch.

### 2.6. Uncertainty analysis.

The uncertainties in measuring instruments are estimated using Eq. 19^90^:

19 $${\mathcal{W}}_{r} = \left[ {\left( {\frac{\partial R}{{\partial x_{1} }}{\mathcal{W}}_{1} } \right)^{2} + \left( {\frac{\partial R}{{\partial x_{2} }}{\mathcal{W}}_{2} } \right)^{2} + \ldots + \left( {\frac{\partial R}{{\partial x_{3} }}{\mathcal{W}}_{3} } \right)^{2} } \right]^{1/2}$$

The initial uncertainty in the measurement of MC, moisture ratio, and drying rate was approximately 1.12%. Additionally, the study assessed potential measurement errors in temperature, relative humidity, wind speed, and solar radiation, which were found to be 0.32%, 0.28%, 0.24%, and 0.13%, respectively. Taking these variables into account, the overall uncertainty in evaluating the efficiency of the HSD was estimated at about ± 2%.

## Results and discussions

3.1. Accumulated weight loss during the drying process of pomegranate peels.

**Fig. 3:**
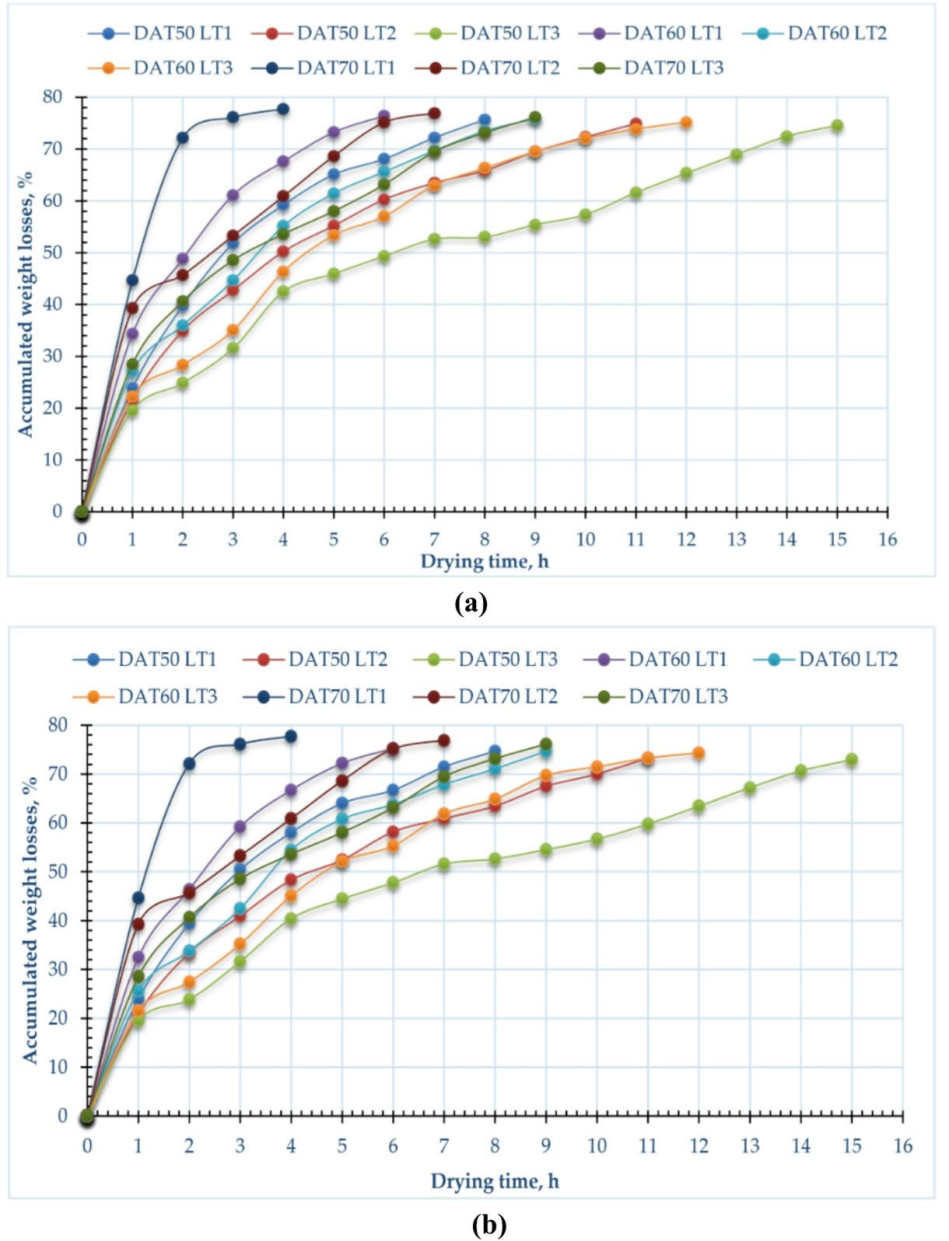
Accumulated weight losses of pomegranate peels at different drying temperatures and layers thicknesses, (**a**) CODS, and (**b**) HISD.

Certain agricultural products experience significant weight loss during processing, which negatively impacts their quality, appearance, and market value. This can manifest as undesirable changes in texture, shape, or color^91^. A major reason for this weight reduction is the leaching and diffusion of water-soluble components—such as vitamins, flavors, minerals, carbohydrates, sugars, and proteins—from the plant tissue into the surrounding environment^92^. Figure 3 presents the cumulative weight loss of pomegranate peels under various drying temperatures and layer thicknesses for both drying methods. The data indicate that there was no significant difference in weight loss between the two drying systems. However, weight loss increased as the drying temperature rose, with the greatest loss observed at 70 °C and the least at 50 °C. This trend suggests that weight loss is directly related to the drying power; the higher the temperature, the greater the weight reduction. Similar observations have been reported by Kidmose and Kaack^93^ and Wang et al.^91^. Furthermore, Fig. 3 shows that there was an inverse relation between weight loss and layer thickness, where it was found that the highest rates of removed moisture were recorded with samples with a thickness of 3 cm, while the lowest rates were recorded with samples with a thickness of 1 cm.

### 3.2. MC

**Fig. 4:**
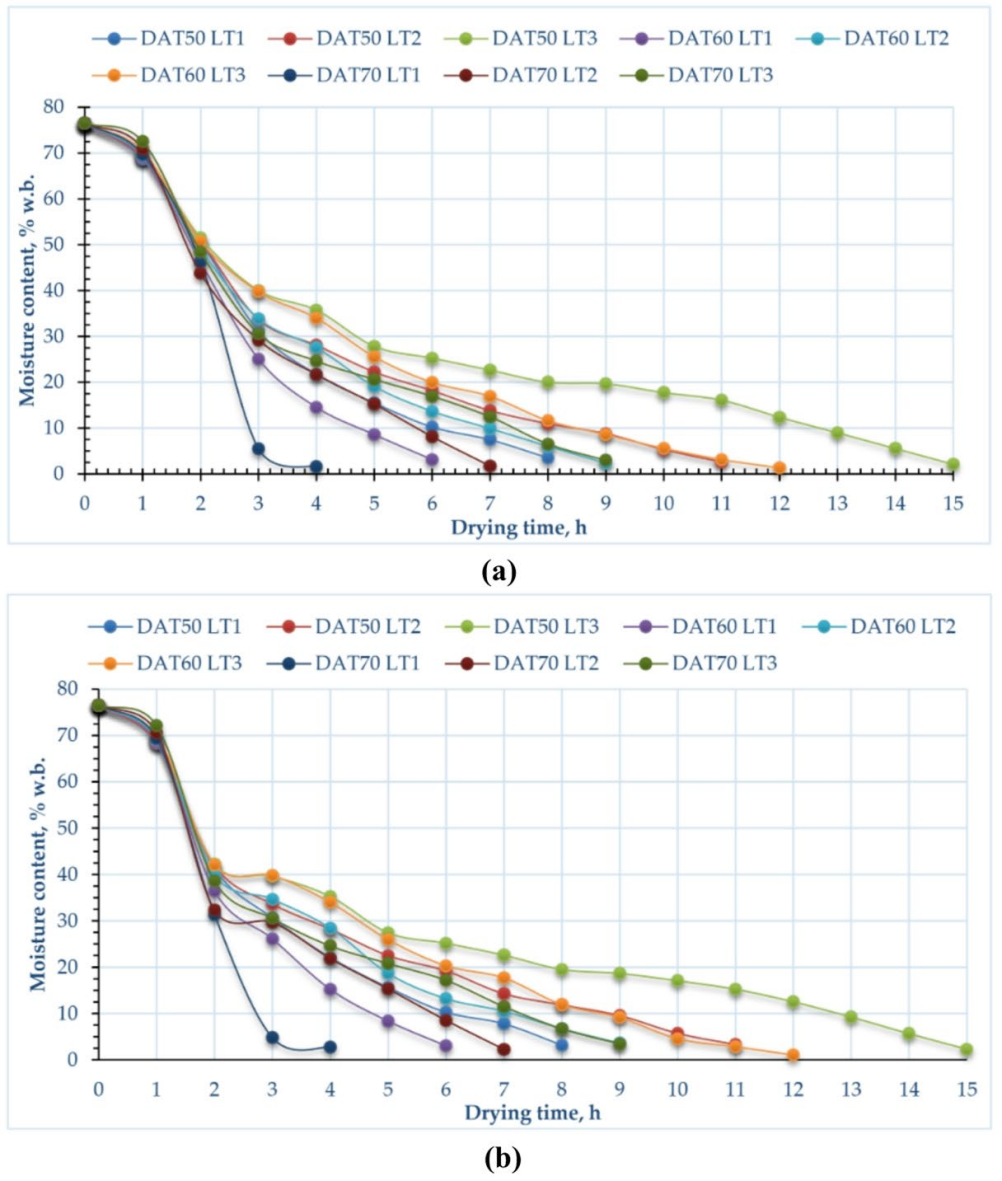
MC on wet basis of pomegranate peels at different drying temperatures and layers thicknesses, (**a**) CODS, and (**b**) HISD.

Figure 4 illustrates how drying methods, layer thicknesses, and drying temperatures affect the MC of pomegranate peels. The average initial MC of pomegranate peels is 76.5% (W.b.). The drying process using an oven dryer led to a decrease in the MC of all pomegranate peel samples from 76.5% to an average MC of 2.67%, 2.27%, and 2.10% at drying temperatures of 50, 60, and 70 °C, respectively. Additionally, the average MC of all pomegranate peel samples dried using HISD was 2.97, 2.56, and 2.84% at drying temperatures of 50, 60, and 70 °C, respectively. On the other hand, the drying process using an oven led to a decrease in the MC of all pomegranate peel samples from 76.5% to an average MC of 2.74, 2.23, and 2.13% at layer thicknesses of 1, 2, and 3 cm, respectively. Furthermore, drying of pomegranate peel using HISD led to a decrease in the MC up to 2.99, 3.07, and 2.27% at layer thicknesses of 1, 2, and 3 cm, respectively. Furthermore, Fig. 4 illustrates that increasing the air temperature from 50 to 70 °C results in a decrease in the drying time of the finished product by about 100%, 57%, and 67% for layer thicknesses of 1 cm, 2 cm, and 3 cm, respectively, across both drying techniques.

### Drying rate

**Fig. 5:**
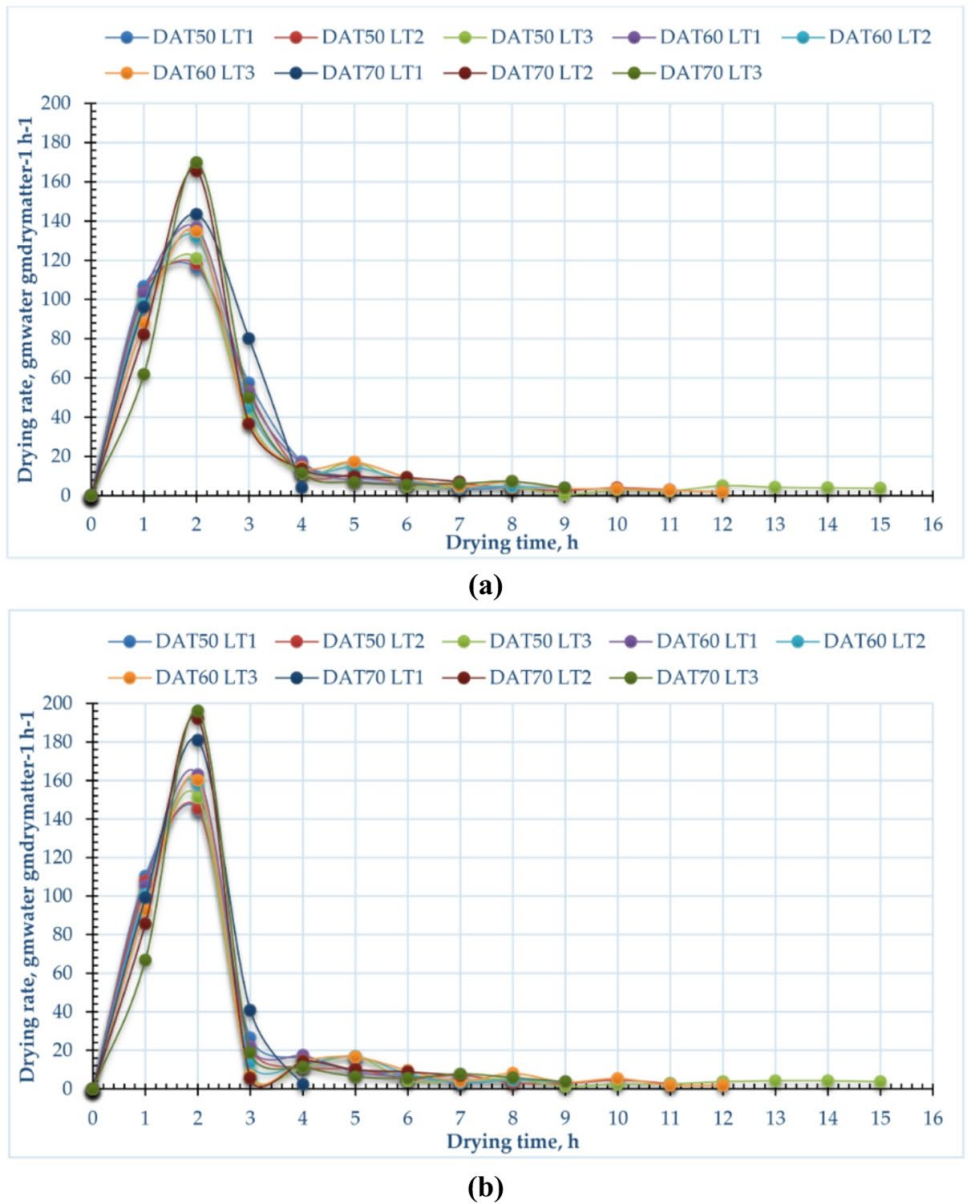
Relation between drying rate and drying time of pomegranate peels at different drying temperatures and layers thicknesses, (**a**) CODS, and (**b**) HISD.

**Fig. 6:**
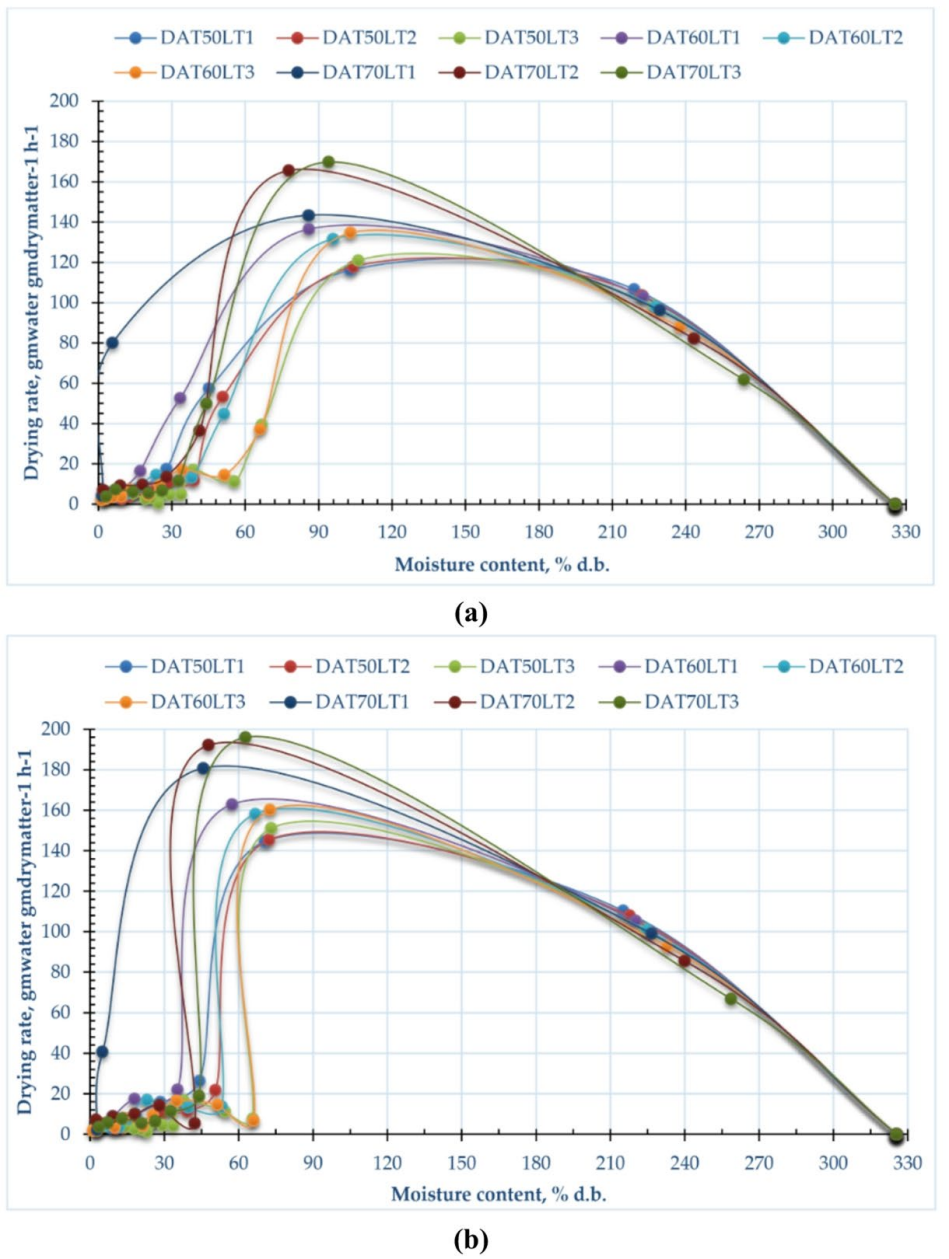
Relation between drying rate and MC of pomegranate peels at different drying temperatures and layers thicknesses, (**a**) CODS, and (**b**) HISD.

Figure 5 illustrates the variance in drying rate concerning time across both drying methods, layer thicknesses and drying temperatures. Also, Fig. 6 shows the relation between the MC and the drying rate at different layer thicknesses and drying temperatures for both drying methods. The higher drying rates of the pomegranate peels dried using CODS ranged between 116.11 to 120.88 kg_water_/kg_dry matter_/h, 136.51 to 134.61 kg_water_/kg_dry matter_/h, and 143.37 to 169.79 kg_water_/kg_dry matter_/h when the layer thicknesses ranged between 1 to 3 cm, for drying temperatures of 50, 60, and 70 ᵒC, respectively. While the higher drying rates of the pomegranate peels dried using the HISD ranged between 144.5 to 151.1 kg_water_/kg_dry matter_/h, 162.9 to 160.1 kg_water_/kg_dry matter_/h, and 180.8 to 196 kg_water_/kg_dry matter_/h when the layer thicknesses ranged between 1 to 3 cm, for drying temperatures of 50, 60, and 70 °C, respectively. Where the plotted data in Fig. 5 showed that there is a direct relation between the drying rate and temperature also, between the drying rate and layer thickness, where the highest drying rate was observed at a layer thickness of 3 cm and drying temperature of 70 °C. The drying rate is significantly influenced by both drying temperature and layer thickness. Higher drying temperatures accelerate the rate by providing more thermal energy, which enhances moisture evaporation and internal diffusion. In contrast, thicker layers slow drying because heat transfer and moisture diffusion to the surface become less effective. Thus, balancing temperature and layer thickness is key: high temperatures can harm heat-sensitive materials, while thick layers cause uneven, prolonged drying^94^. Similar results were reported by Darvishi et al.^95^, showing that drying rates decline over time as moisture content drops. The highest drying rate occurs early, during free moisture removal, then steadily decreases. Most of the drying takes place in the falling-rate period, with a short initial constant-rate phase. Internal mass transfer resistance mainly determines drying duration. As noted by Elshehawy et al.^94^, moisture ratio reduction is slower at the start and faster toward the end. Table 3 shows the summary of previous studies on pomegranate peel drying.

**Table 3:** Summary of previous studies on pomegranate peel drying.

Refs.	Drying method	Temp ( °C)	Air velocity (m/s)	Time (h)	Initial–final moisture (g/g d.b.)	Drying rate (g _water_/g_drymatter_·h)
^67^	Tray dryer (Oven)	50	Not stated	14	0.1344 (equilibrium)	0.0096
		60		13	0.0909	0.0070
		70		12	0.0770	0.0064
^96^	Hot air dryer (Oven)	50	2.0	~ 5	~ 2.44 → 0.044	~ 0.480
^97^	Forced convection dryer (Oven)	65	1.0–3.0	3–4	2.44 → ~ 0.05	0.598–0.798

### 3.4. Power consumption.

Table 4 compares power consumption for drying pomegranate peels using CODS and HISD. Where the power consumption is measured at different thicknesses (1 cm, 2 cm, and 3 cm) and temperatures (50 °C, 60 °C, and 70 °C). The findings show that, in the CODS system, the highest power consumption occurred at 50 °C with a product thickness of 1 cm, reaching 7769 W kg⁻^1^, while the lowest was recorded at 70 °C with a 3 cm thickness, measuring 3453.7 W kg⁻^1^. Similarly, in the HSD system, the highest power usage was also at 50 °C and 1 cm thickness, at 2116.4 W kg⁻^1^, whereas the lowest was observed at 70 °C and 3 cm thickness, at 961.4 W kg⁻^1^. Where the tabulated data indicated that power consumption was decreasing with increasing the drying temperature. Additionally, it decreased with increasing layer thickness. where the total power consumption for drying is influenced by both layer thickness and drying temperature. Higher drying temperatures typically increase power consumption due to the greater power required to generate and sustain the heat. However, this can be offset by faster drying rates, potentially reducing overall power use if drying time is significantly shortened. Thicker layers, on the other hand, generally increase total power consumption because more power is needed to penetrate and dry the additional material mass, often extending the drying duration. Therefore, optimizing both layer thickness and drying temperature is crucial to balance power efficiency and drying effectiveness, minimizing total power consumption while ensuring quality results.

**Table 4:** Power consumption for drying pomegranate peels using CODS and HISD under different drying air temperatures and different layers thicknesses.

Energy consumption, W kg^−1^				
	Layer thickness, cm	50 °C	60 °C	70 °C
CODS	1	7769	6571.1	4662.1
	2	4939.6	4141.1	3932.9
	3	4073.9	3775.1	3453.7
HISD	1	2116.4	1479.1	1350.9
	2	1371.5	1009.8	998.7
	3	987.9	978.7	961.4

Table 5 shows the reduction percentage of power consumption for drying pomegranate peels by using the HISD compared to CODS. Where the data presented showed that the use of HISD led to a reduction the power consumption by about 64.28% to 75.75%. Hybrid SDs provide significant economic and financial advantages over conventional oven dryers. By harnessing free solar energy as the primary heat source, they drastically reduce energy costs and dependence on expensive fossil fuels. Although the initial investment may be higher, the long-term savings in operational and maintenance costs make them highly cost-effective. They also have lower running expenses due to minimal energy consumption and can operate in off-grid areas, reducing infrastructure costs. Table 6 shows the power consumption for drying pomegranate peels.

**Table 5:** Reduction percentage of power consumption by using the HISD compared CODS.

Layer thickness, cm	50 °C	60 °C	70 °C
1	72.75%	77.49%	71.02%
2	72.23%	64.28%	74.61%
3	75.75%	74.08%	72.16%

**Table 6:** Power consumption for drying pomegranate peels.

Drying method	Power/conditions	Specific energy consumption (SEC)	Refs.
Microwave vacuum drying	260–287 W, 15–19 kPa	20.01–20.02 kWh/kg	^98^
Microwave vacuum drying	276 W, 10 kPa (optimized)	Not numerically specified (high efficiency)	^99^
Infrared/convection drying	150–250 W, 40 °C, 1.5 m/s air	Not specified in abstract	^100^

### Environmental analysis

**Fig. 7:**
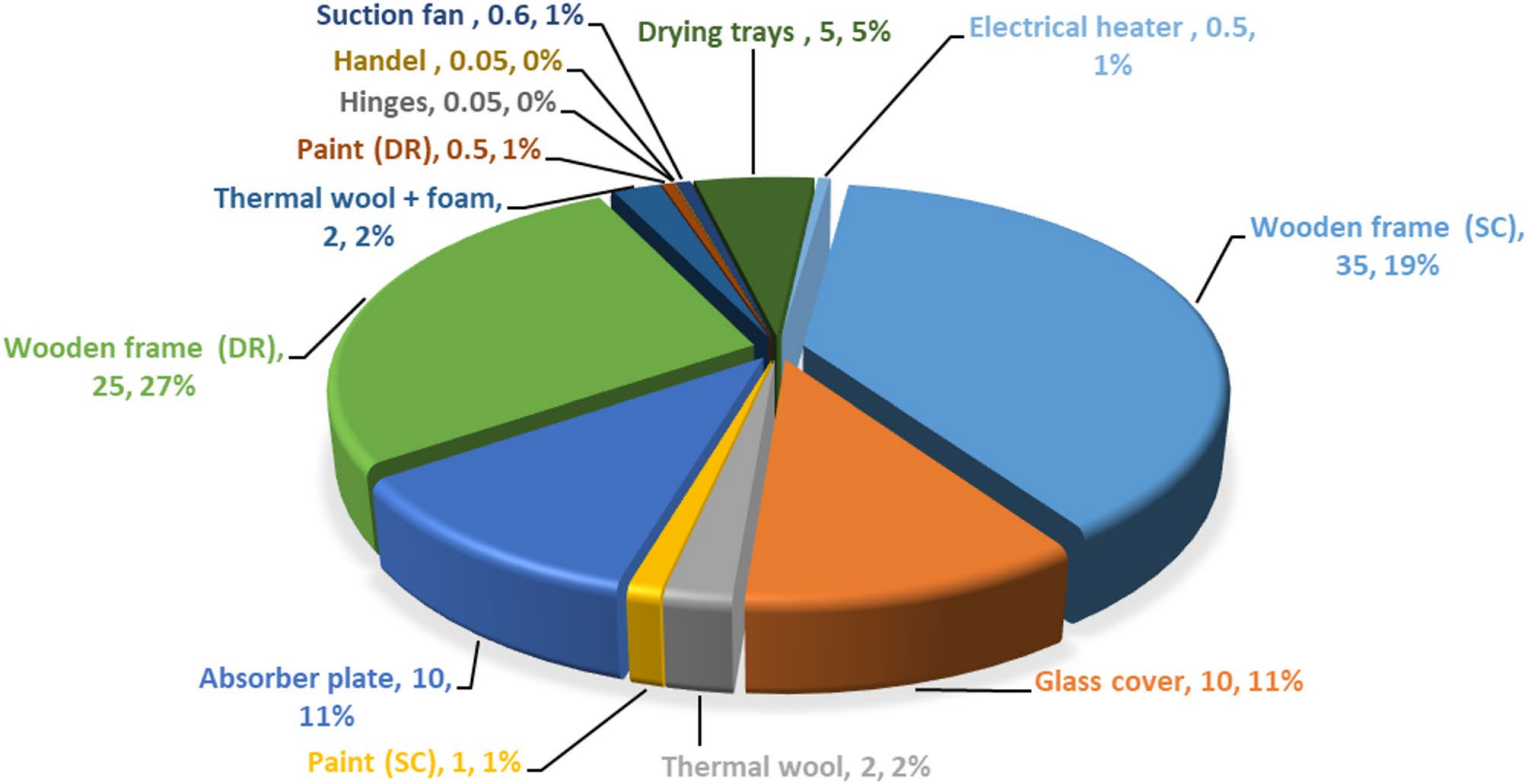
Distribution of the mass of various materials used in building the HISD.

Figure 7 illustrates the mass distribution of materials used in constructing the HISD. The largest portion, 46% (60 kg), corresponds to the wooden structure of both the drying room and the solar collector. Next, the glass cover and absorber plate of the solar collector contribute 22% (20 kg) of the total weight. The remaining weight is shared among various other components, such as insulation materials (thermal wool and foam), paints, drying trays, electric heater, air suction fan, and the door’s handle and hinges.

**Fig. 8:**
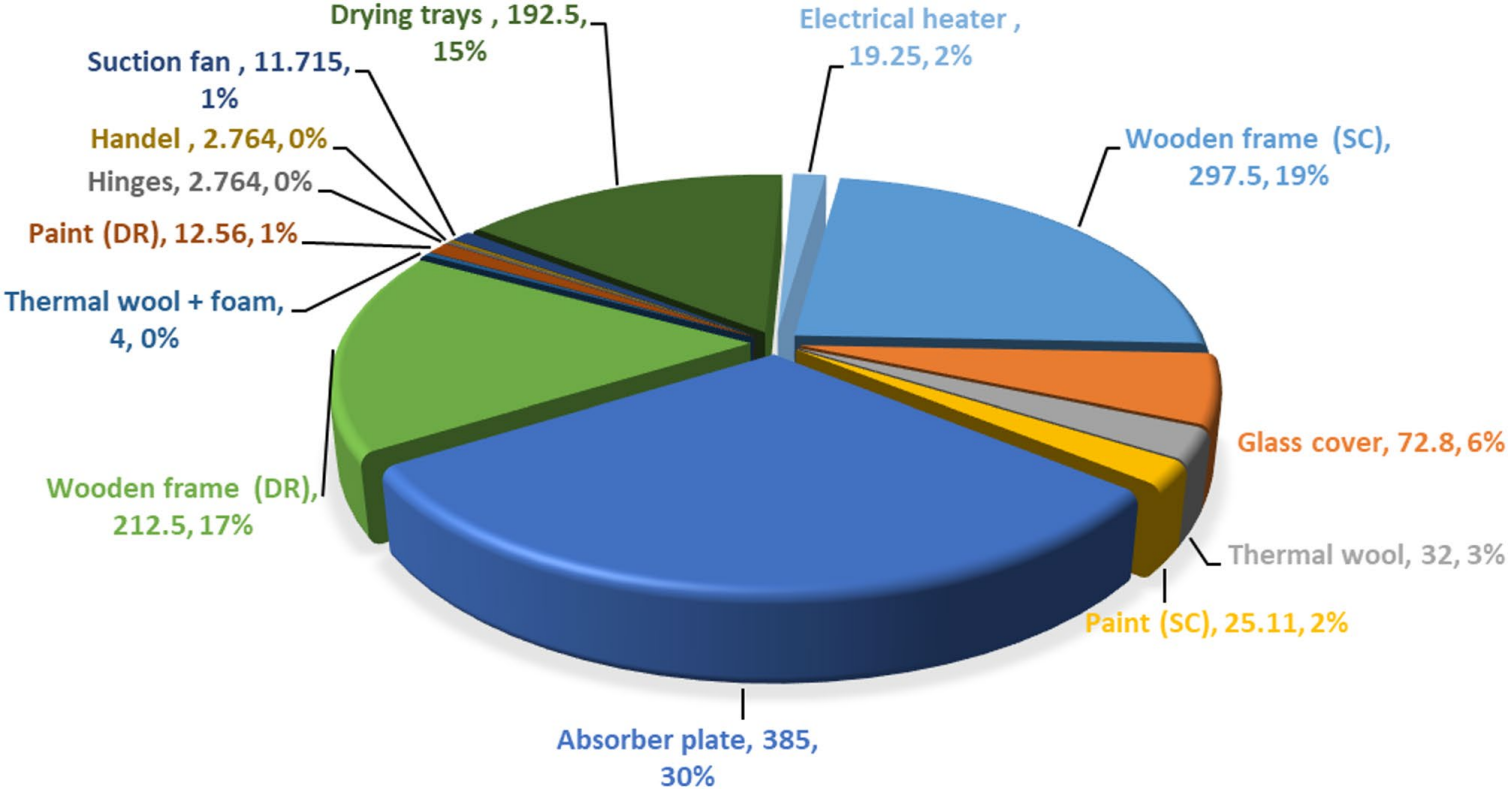
Distribution of EE among the various materials used in constructing the HISD.

Figure 8 displays a comprehensive breakdown of the distribution of EE among various components utilized in the manufacturing of the HISD. The EE is about 1270.463 kW.h. The wooden frame represented the major value of the EE, amounting to 36% of total EE (510 kW.h). Followed by the absorber plate which represents 30% of the total EE. Additionally, the drying trays come in third place, where it represented about 15% of the total EE. Furthermore, the remaining EE percentages, about 19%, are distributed among other components, including the thermal wool and foam (insulation materials), paints, drying trays, electrical heater, air suction fan, handel and hinges of the drying room’s door.

The dryer is operational for 10 h every day, from 07:00 to 17:00. The value assigned to the latent heat of vaporizing water is 2257 kJ/kg. The environmental factors were analyzed using Eqs. 4–13 over 30 years, taking into account drying methods, layer thicknesses, and drying temperatures. The results can be seen in Table 7. Table 7 shows that the annual carbon dioxide emissions and mitigation were approximately 41.5 kg and 75.5 tons, respectively. Additionally, the energy payback period for the HISD ranges between 2.38 and 6.34 years. Drying the pomegranate peels at a drying temperature of 70 °C and layer thicknesses of 2 cm and 3 cm will result in a shortened energy payback period. The drying of pomegranate peels at a temperature of 50 °C and a layer thickness of 1 cm will result in the longest energy payback period. The current duration is significantly less in comparison to the lifespan of the HISD (30 years). The net CO_₂_ mitigation over the lifetime achieved by using HISD for various durations is presented in Table 4. The earned carbon credit is USD 72.50 per metric ton of CO_2._ As shown in the same table, the earned carbon credit for drying pomegranate peels using the HISD ranged between 770.1 and 2207.2 USD. Table 8 shows the comparison with some previous studies.

**Table 7:** Environmental analysis for the HISD at different layer thicknesses and drying temperatures.

Economic analysis	Layer thickness, cm & drying temperature,** °**C								
	50 °C			60 °C			70 °C		
	1 cm	2 cm	3 cm	1 cm	2 cm	3 cm	1 cm	2 cm	3 cm
CO_2_ emission (Kg year^−1^)	41.5	41.5	41.5	41.5	41.5	41.5	41.5	41.5	41.5
CO_2_ mitigation (tons)	75.5	75.5	75.5	75.5	75.5	75.5	75.5	75.5	75.5
Annual thermal output (kJ year^−1^)	200.24	291.25	320.38	266.98	355.97	400.47	400.47	533.96	533.96
Net CO_2_ mitigation over the lifetime (tons)	10.6	16	17.8	14.6	19.9	22.5	22.5	30.4	30.4
Energy payback period (years)	6.34	4.36	3.97	4.76	3.57	3.17	3.17	2.38	2.38
Earned carbon credit (USD)	770.1	1162	1287.5	1057.5	1440.7	1632.4	1632.4	2207.2	2207.2

**Table 8:** Comparison with some previous studies.

Study/system type	Energy payback period (years)	Key operating conditions/features	Environmental impact highlights	Refs.
Large scale SD with PCM energy storage	6.82	Industrial scale, PCM storage, focus on exergy and cost analysis	CO₂ mitigation: 99.6 tons over system lifetime	^101^
Domestic direct-type multi-shelf SD	7.57	Multi-shelf, no load, CFD validated, 326 K air temp	Carbon credit: INR 2055	^102^
Greenhouse SD (Quonset, parabolic roof)	3.1 (EPBT), 1.64 (PBP)	Polycarbonate, 2.47 × 6.0 m^2^, 2 fans, 30–48 °C outlet temp	Net CO₂ mitigation: 130.7 tons	^103^
Indirect SD with PCM (aluminum flat coil)	1.44	Paraffin wax PCM, summer, 10.25 h drying, 21% thermal efficiency	CO₂ mitigation: 20.12 tons over 35 years	^104^
Automatic SD for date fruits	7.54–7.71	Automated, thin-layer, 9–10 days drying, 17.9–22.7 kWh/kg energy use	Net CO₂ mitigation: 8.55–8.80 tons	^105^
Indirect cocoa bean SD (forced/natural conv.)	2.19	Forced convection day, natural night, 32 h drying, 5–15 kWh/kg	CO₂ mitigation: 15–25 g/kg water evaporated	^106^
HISD	2.38	Layer thickness of 3 cm & drying temperature 70 ° C	CO₂ mitigation: 75.5 tons	Current study

### Economic analysis

The economic analysis in this study searched for a way to combine the HISD with an axillary electrical heater that would benefit the economy. The study utilized Eqs. 14–18, which addressed the life cycle savings technique and the simple payback methodology. Tables 9 and 10 illustrate some of the key considerations. The current state of the Egyptian economy and the expected costs of the HISD components were also looked at. As shown in Table 9, the capital cost of the HISD is very low compared with conventional industrial dryers, where it was only about 200 USD. This low capital cost led to a decrease in the annualized investment cost and reduced the drying cost per ton of dried pomegranate peels. As shown in Table 4, the power consumption for drying pomegranate peels decreased with increasing the drying temperature. Additionally, it decreased with increasing layer thickness. Additionally, the drying cost per ton of pomegranate peels was calculated based on Eqs. 13–15, and the obtained results were tabulated in Table 6. In Table 6, the bold numbers in colored boxes represented the lowest drying costs for both drying methods at different layer thicknesses and drying temperatures. It can be concluded that the lowest drying costs were 144.5 USD per ton of pomegranate peels, achieved at layer thicknesses of 1 cm and a drying temperature of 70 °C, where the drying time is the lowest compared to other layer thicknesses and the annually dried qualities of pomegranate peels are the highest compared to other layer thicknesses.

**Table 9:** Various costs related to the HISD.

Cost parameters	HISD
Capital cost, USD	200
Annual capital cost, USD	21.22
Annual maintenance cost, USD	0.64
Annual salvage value, USD	1.7
Annualized investment cost, USD	21.84

**Table 10:** Economic analysis of both drying systems for drying pomegranate peels.

Economic analysis		Layer thickness, cm & drying temperature, °C								
		50 °C			60 °C			70 °C		
		1 cm	2 cm	3 cm	1 cm	2 cm	3 cm	1 cm	2 cm	3 cm
CODS	Annually dried quantity, kg year^**−1**^	131.3	95.5	70	175	116.7	87.5	262.5	150	116.7
	Drying cost, USD ton^-1^	352.7	224.3	185	298.3	188	171.39	211.7	178.6	156.8
HISD	Annually dried quantity, kg year^−1^	131.25	95.5	70	175	116.7	87.5	262.5	150	116.7
	Drying cost, USD ton^−1^	262.5	291.1	356.9	192	233.1	273.3	144.5	206.9	203.9

## Conclusions

In this study, pomegranate peels were dried in a HISD with a temperature and humidity control unit and a CODS. The peels were dried at 50 °C, 60 °C, and 70 °C, and they were cut into three layers that were 1, 2, and 3 cm thick. The results obtained showed that the weight loss of pomegranate peels increased by increasing the drying temperature. Also, the average initial MC of pomegranate peels was 76.5% on a wet basis (w.b.). The final MC reached 2.10% and 2.84% for the CODS and HISD, respectively. The higher drying rates of the pomegranate peels dried using CODS and HISD were 169.79 and 196 kg_water_/kg_drymatter_/h, respectively, at a layer thickness of 3 cm and a drying temperature of 70 °C. Additionally, using HISD led to a reduction in power consumption by about 64.28% to 75.75% compared to the CODS. Furthermore, the environmental analysis results showed that the cumulative energy required for the established system (EE) is about 1270.463 kW.h. Where the carbon dioxide emissions and mitigation were about 41.5 kg year − 1 and 75.5 tons, respectively. The energy payback period for HISD ranges between 2.38 and 6.34 years. The shortened energy payback period can be achieved by drying the pomegranate peels at a drying temperature of 70 °C and layer thicknesses of 2 cm and 3 cm. The earned carbon credit for drying pomegranate peels using the HISD ranged between 770.1 and 2207.2 USD. Based on economic analysis, the capital cost of the HISD is very low compared with conventional industrial dryers, where it was only about 200 USD. This low capital cost led to a decrease in the annualized investment cost and reduced the drying cost per ton of dried pomegranate peels. Moreover, the power consumption for drying pomegranate peels was decreasing with the increasing temperature. Additionally, it decreased with increasing layer thickness. It can be concluded that the lowest drying costs at all drying temperatures were achieved at layer thicknesses of 1 cm, where the drying time is the lowest compared to other layer thicknesses and the annually dried qualities of pomegranate peels are the highest compared to other layer thicknesses.

### Limitation of the current study

The study primarily focused on drying kinetics, energy use, and microbial safety. However, the impact of drying on bioactive compound retention, antioxidant activity, or functional properties of the dried peel was not deeply explored.

## Data Availability

All data are provided within the article.
